# Is mental training suitable for teaching a surgical procedure to students? A single-center study using a hernia model

**DOI:** 10.1007/s10029-025-03466-w

**Published:** 2025-10-03

**Authors:** Miro Kopp, Guido Woeste, Hanan El Youzouri, Ursula Pession, Jasmina Sterz, Miriam Ruesseler, Wolf O. Bechstein, Teresa Schreckenbach

**Affiliations:** 1 Goethe-University Frankfurt/Main Frankfurt, Department of General, Visceral, Transplantation, and Thoracic Surgery, University Hospital and Clinics, Theodor‑Stern‑Kai 7, 60590 Frankfurt/Main, Germany; 2 Department of General and Visceral Surgery, AGAPLESION Elisabethenstift, Landgraf-Georg-Str. 100, 64287 Darmstadt, Germany; 3 Medical faculty, Institute for medical education and clinical simulation, Goethe University Frankfurt/Main, Theodor Stern Kai 7, 60590 Frankfurt/Main, Germany; 4 Goethe‑University Frankfurt/Main, Department of Trauma, Hand and Reconstructive Surgery, University Hospital and Clinics, Theodor‑Stern‑Kai 7, 60590 Frankfurt/Main, Germany

**Keywords:** Abdominal wall hernia, Mental skills training, Medical students, Benchtop models, Medical education

## Abstract

**Purpose:**

New methods of teaching surgical skills are in demand. Mental skills training (MST) has been proven effective in the training of surgeons. However, research on medical students is still rare. This explorative study investigates whether a minimalist form of MST can support surgical performance on a hernia model in medical students in a way comparable to students trained using a conventional ‘see one, do one’ approach.

**Methods:**

A novel inexpensive benchtop training model for abdominal wall hernias has been developed. Medical students were randomized into an ‘See one, do one’ and a ‘See one’ + MST group. Both groups received the same theoretical instructions, after which a tutor demonstrated the operation on the model. While the first group received training in MST afterwards, the ‘See one, do one’ group was trained using the benchtop model. Subsequently, both groups performed surgery on the model. Their performance was videotaped and evaluated by experienced surgeons.

**Results:**

A total of 44 medical students took part in the study. The ‘See one, do one’ group performed significantly better in the professional examination, although the consistency of the stitching was comparable between the two groups and showed no statistically significant differences.

**Conclusion:**

‘See one’ + MST group alone without practical training did not result in comparable performance as the ‘See one, do one’ approach in our study. The MST group performed inferiorly across most objective metrics. While this suggest that MST in isolation may not be sufficient for teaching complex procedures to medical students, it highlights the importance of practical exposure. The potential benefit of combining MST with hands-on training should be investigated in future studies, but cannot be inferred from the current data.

**Supplementary Information:**

The online version contains supplementary material available at 10.1007/s10029-025-03466-w.

## Introduction

### Background

Today, many medical students feel unprepared for residency training [1]. Especially in the surgical disciplines, many young colleagues report that their medical school curriculum did not adequately prepare them for everyday working life [2]. Established methods, such as Halsted’s ‘see one, do one, teach on’ or ‘learning by doing’ has lost its significance due to patient safety concerns [3, 4]. Therefore, innovative teaching methods e.g. video-based approaches, and mental skills training (MST) have become the subject of current research [5–7].

MST encompasses a variety of cognitive strategies aimed at enhancing motor performance, skill acquisition, and stress management in clinical and surgical education [8]. These strategies include mental imagery, mental rehearsal, visuomotor training, and cognitive task simulation, which differ in structure, theoretical foundation, and application context [9]. One of the most widely studied approaches is mental imagery, which involves the internal simulation of a task without overt physical movement, typically engaging both visual and kinaesthetic components of the experience [10, 11].

The PETTLEP model (Physical, Environment, Task, Timing, Learning, Emotion, Perspective) has been proposed as a framework to guide imagery practice, emphasizing functional equivalence between imagined and real-world performance [12]. In contrast, brief, guided mental rehearsal sessions, even when not explicitly PETTLEP-based, have demonstrated significant short-term benefits in novice learners, especially under time-limited educational conditions [13, 14].

MST describes the process of mentally visualizing a movement to positively influence its execution [9, 15]. The neurophysiological backgrounds have been well established, by showing a functional relationship between motor images and their corresponding motor representation [16]. MST is widely used as a tool in sports psychology, especially in precision sports [9, 17]. The integration of MST into surgical training mainly serves two purposes. Firstly, it is used as a visualization tool, practicing movements and improving motor performance [7]. Secondly, it is used as a stress management tool, keeping surgical performance consistent under stressful situations [18, 19]. Immenroth et al. showed that MST can be especially effective when paired with practical training [6]. Based on this, Mayer and Hermann formulated five steps for integrating MST into surgical training [9]:

1. Choosing the type of surgery
2. Describing the procedure
3. Instructing (condensing the steps into several bullet points)
4. Combining practical training and MST
5. Executing

MST represents a cost-effective and risk-free method to support surgical training without exposing patients to harm. It can be implemented in various settings, including preoperative preparation, dedicated training sessions, and independent practice at home [8]. While the effectiveness of MST has been studied in professional surgeons, particularly those aiming to optimize their performance, its application in medical education remains relatively underexplored [6, 18, 20]. Existing studies suggest that MST may support the acquisition of isolated surgical skills in medical students; however, the current evidence base is sparse and lacks consistency [21, 22]. It remains unclear, whether MST can facilitate the learning of complete and more complex surgical procedures – such as the repair of abdominal wall hernia- in novice learners [10, 23]. Given that the active performance of surgical procedures, even on simplified models, may enhance students’ understanding of fundamental surgical principles and procedural logic, further investigation into the integration of MST in medical students surgical education is warranted.

### Aim of the study

This prospective, randomized and blinded study aimed to analyse whether MST can support the learning process and execution of a more complex surgical procedure in a way comparable to the ‘See one, do one’ approach using the example of the treatment of an abdominal wall hernia on a training model. Students who had received MST were compared to those who had trained practically with a training model. Different aspects of surgical performance were measured and compared: speed, accuracy, technique, and knowledge of the surgical steps. This study tested a minimalist version of MST (a single 45-minutes session without feedback or repetition) and should be considered an exploratory assessment rather than a formal non-inferiority trial.

## Materials and methods

This prospective, blinded and randomized study was reviewed by the Ethics Committee of the University Hospital in Frankfurt (Goethe University), which indicated that no further approval was needed (No. 2023-1228).

### Study participants

Study participants had been recruited during surgical rotations and via social media. They received a detailed oral and written explanation of the study and provided written informed consent. Participation in the study was voluntary and could be terminated at any time. Participants received no monetary compensation for their participation.

The inclusion criteria comprised students in their 3rd to 5th year of study who voluntarily participated in the study and provided written informed consent. The exclusion criteria were previous training, such as scrub nurse, other formal surgical training, or having already completed the fifth year of medical school.

Students filled in a questionnaire about descriptive data such as age and year of medical school, their previous surgical knowledge and their interest in pursuing a surgical field.

### Model

**Fig. 1:**
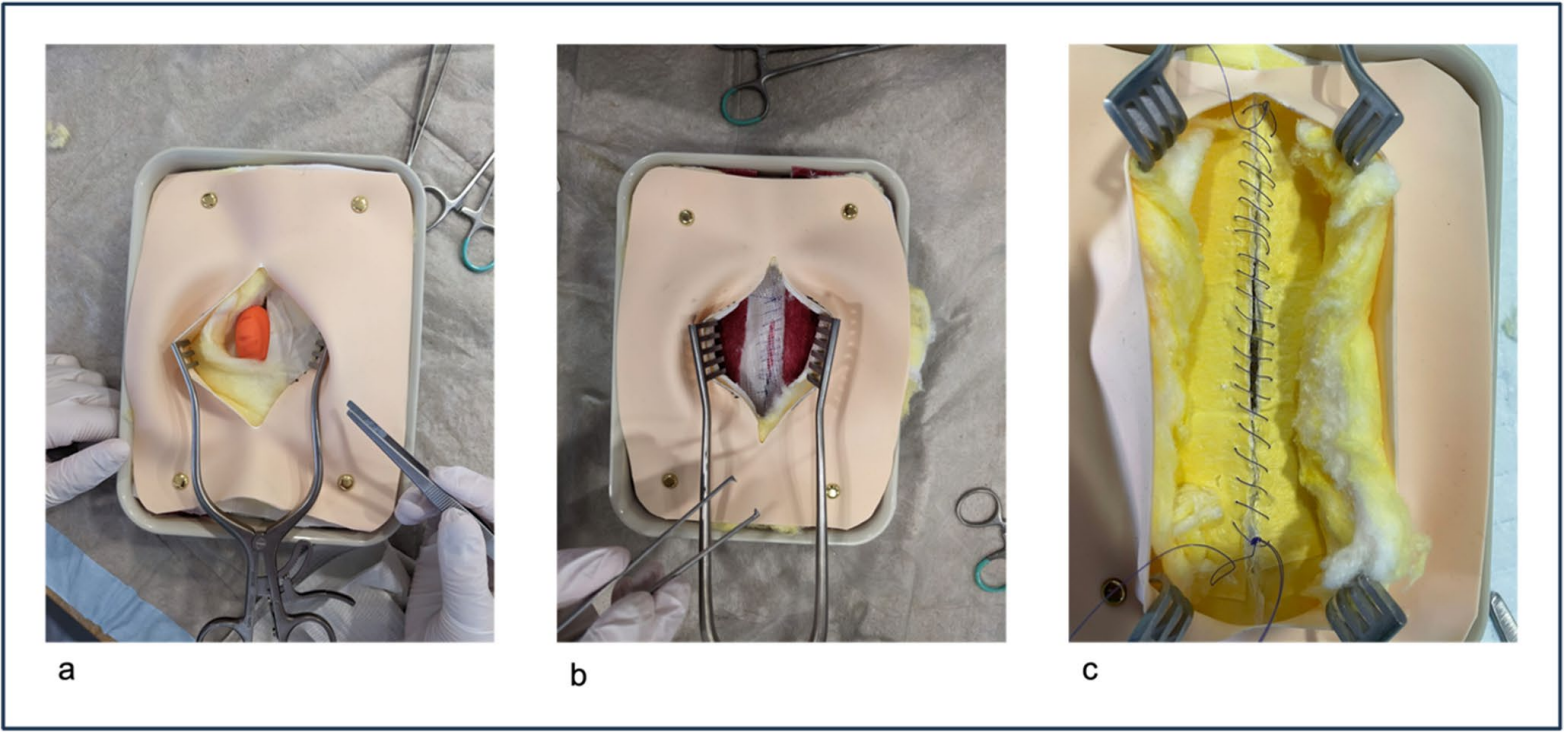
Stepwise representation of the abdominal wall hernia training model during simulated repair: (**a**) Skin and subcutaneous tissue have been incised and retracted to expose the hernia sac (**b**) The posterior rectus sheath is opened and closed with a running suture; a mesh is placed in the retromuscular space. The anterior rectus sheath remains open and is visible through the wound retractor. (**c**) The anterior rectus sheath is closed using a continuous “small stitches, small bites” technique to simulate fascial closure

During the first phase of the study, we constructed a novel benchtop training model to demonstrate abdominal wall hernia surgery (Fig. 1). This model was composed of readily available materials sourced from hardware and arts supply stores, including silicone sheets, foam rubber, fleece and cotton wool. A wool-filled balloon served as hernia sac and a mosquito net was used to simulate mesh placement. The model was developed to replicate the anatomical layers involved in a typical Rives-Stoppa repair. It consisted of multiple structured layers, arranged from deep to superficial as follows: a sponge-like base layer as posterior support, a sheet of reinforced foam rubber (posterior fascia), a red sponge layer (representing the rectus abdominis muscle), another foam rubber layer (anterior fascia), synthetic snow panels or cotton wool (subcutaneous fat), and tattooing practice skin to mimic the dermal surface. The hernia sac was embedded between the fascial layers to allow for realistic dissection and preparation. All layers were connected in a way that permitted tissue preparation, especially for creating a retromuscular space suitable for mesh positioning.

Although the model simplified certain anatomical aspects, it allowed students to practice all major components of open hernia surgery, including skin incision, tissue dissection, fascial suture, retromuscular mesh placement, and closure techniques. The model design was based on core surgical steps described in a standard hernia surgery reference work, and tailored for educational purposes [24].

An important limitation is that the foam-based fascia layers are relatively rigid, which allows for suture placement but does not permit full tissue adaptation, as shown in Fig. 1c. As a result, mesh overlap had to be Limited to 1.5 cm, and the defect in the anterior fascia at the linea alba had to be surgically enlarged to ensure sufficient exposure of the muscle layer and posterior fascia. Despite these constraints, the model fulfilled its primary purpose as a cost-effective, partially reusable, and easily reproducible platform for procedural training at the student level.

### Structure of MST

The MST followed the five steps of Mayer and Hermann detailed above [9]. Firstly, following the surgical demonstration, participants in the interventional group were asked to collaboratively identify and articulate the key procedural elements of the hernia repair. These were recorded as bullet points on a flipchart. The different groups identified between 14 and 16 steps. Examples of these recorded procedural steps are provided in Supplementary 1.

Secondly, students proceeded to an individual mental practice session. They were instructed to mentally rehearse the previously defined steps using the flipchart as a guide and to engage in vivid mental imagery of the procedure, including spatial orientation, sequence of actions, and instrument handling. To support this process, participants were permitted to observe and touch the demonstrator model as well as to handle the surgical instruments involved in the task. Furthermore, they were allowed to sit in front of their own training model and to physically rehearse the corresponding hand movements and instrument manoeuvres, without making actual contact with the model itself. During this time, could also consult with the instructors for clarification or feedback. After the designated 45 min, the flipchart was removed from the room prior to the execution of the full procedure, as conducted in both study groups.

### Study protocol

**Fig. 2:**
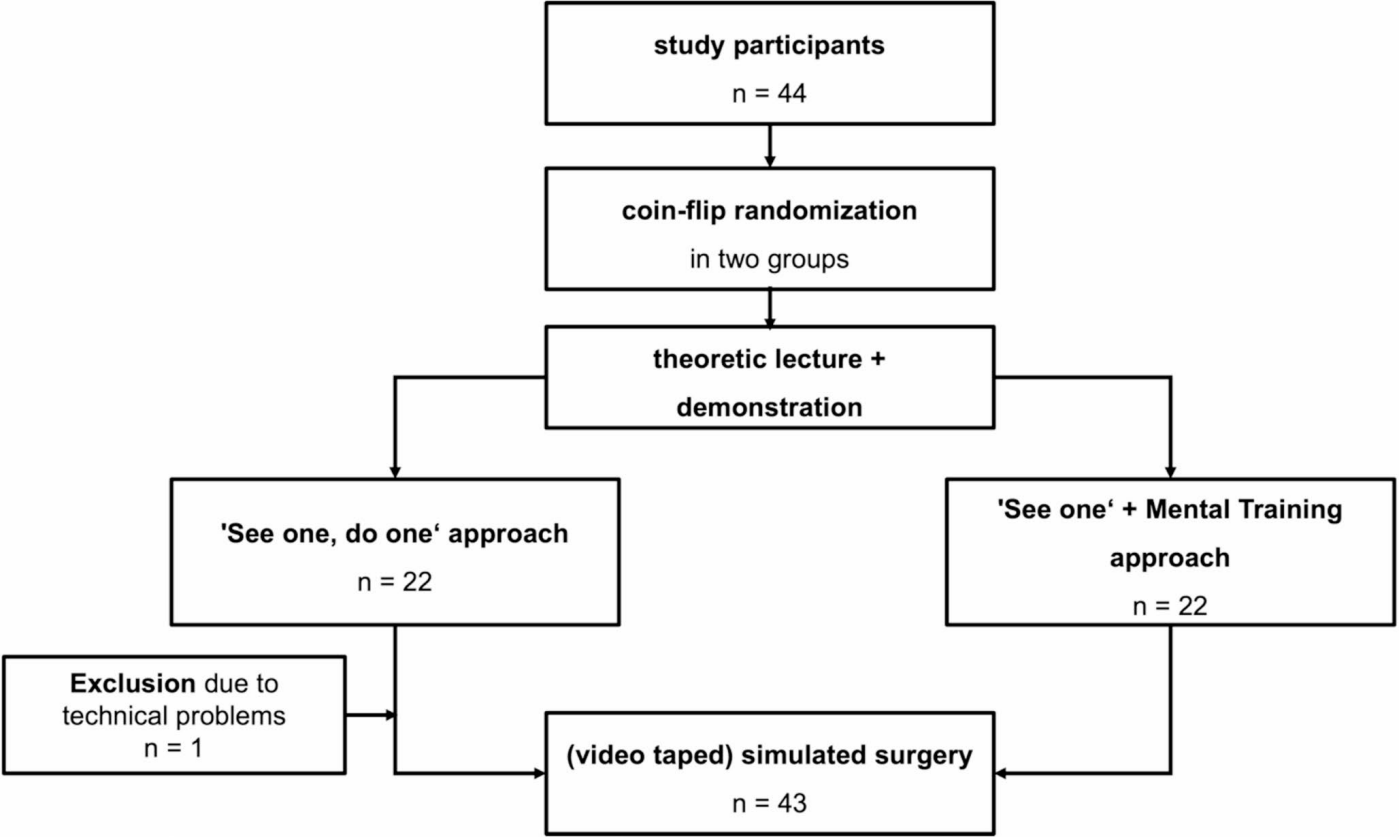
Flow chart of study design and execution

The study was conducted at the University Hospital in Frankfurt/Main. Participant groups were randomized by a coin-flip and assigned into the MST or the conventional group, where they received separate training. Both groups first attended a 20-minute lecture on abdominal wall hernia anatomy, pathophysiology, and therapy. Then, they received a demonstration by one of the instructors (TS) who performed the surgery on the model. The ‘See one, do one’ group received the model to perform the procedure themselves for the training purpose. Simultaneously, the intervention group received a 45-minute training session of MST. Ultimately, both groups performed the surgery on a new model, while being filmed by desktop point of view cameras. The recorded video only showed the model and the students’ hands. Figure 2 shows a flow chart of the study protocol.

### Outcome measures

To assess the performance of the participants, three different factors were evaluated. Firstly, the time needed to perform the surgery was measured. Secondly, the distance between the fascia sutures was measured. Students were instructed to use the ‘small stitches, small bites’ rule, Leaving 5 mm between defect and stitches and between the stitches themselves. This method has been proven most effective for abdominal incisions [25]. The deviation from the 5 mm rule was calculated.

Thirdly, the videos were assessed by experienced surgeons (TS, HEY, UP, GW). They are all board certified, experienced surgeons, all of whom regularly perform this procedure. They were blinded to the assigned groups. The videos were assigned randomly to the evaluators to minimize bias. A structured questionnaire comprising ten distinct evaluation criteria was utilized (Table 2). The questionnaire was developed by an experienced surgeon and specialist in medical didactics (TS). It was subsequently tested for item clarity, questionnaire structure, and content validity by two additional experienced surgeons (UP, HEY). Based on their feedback, the form was revised and refined. Each criterion was scored on a scale from 0 to 2, where 0 indicated that the task was either not performed or performed incorrectly; 1 indicated that it was performed but could have been better executed; and 2 indicated that it was performed optimally. The maximum achievable score was 20. Each participant’s performance was independently assessed by two evaluators, and the final score was calculated as the average of the two ratings. Cronbach’s Alpha of the questionnaire was 0.8.

### Statistics

All statistical analyses were performed using IBM SPSS Statistics for Windows (version 29.0; IBM, Chicago, IL, USA). Categorical variables were described as frequencies and percentages. Continuous variables were presented as the mean and standard deviation (SD). Categorical variables were compared using the chi-squared (χ2)-test. Continuous variables were compared using a one-way analysis of variance (ANOVA). Post-hoc pairwise comparisons were adjusted using the Bonferroni correction to control for multiple testing. A p-value < 0.05 was considered statistically significant.

## Results

In total, 44 medical students participated in this study. Each student filled out a questionnaire on their demographic and previous surgical knowledge (Table 1). There were no significant differences between the two groups, especially regarding the self-assessment of practical skills and suturing technique. Participants in the ‘See one’ + MST group had significantly more previous experience with MST.

**Table 1:** Descriptive statistic of study participants

	Total (%) n = 43	‘See one, do one’ n = 21	‘See one’ + MST n = 22
Gender			
Female	29 (67.4)	13 (61.9)	16 (72.7)
Male	13 (30.3)	7 (33.3)	6 (27.3)
Diverse	1 (2.3)	1 (4.8)	0
Interest in surgery			
Yes	31 (72.1)	16 (76.2)	15 (68.2)
No	3 (7.0)	1 (4.8)	2 (9.1)
Neutral	9 (20.9)	4 (19.0)	5 (22.7)
Vocational training before studying			
None	31 (72.1)	16 (76.2)	15 (68.2)
Medical	6 (14.0)	3 (14.3)	3 (13.6)
Others	6 (14.0)	2 (9.5)	4 (18.2)
Experience with mental training			
Yes	5 (11.6)	0	5 (22.7)
No	38 (88.4)	21 (100)	17 (77.3)
	Total (mean ± SD) n = 43	‘See one, do one’ (mean ± SD) n = 21	‘See one’ + MST (mean ± SD) n = 22
Age in years	23.9 (± 3.4)	24.0 (± 3.8)	23.9 (± 3.1)
Self-assessment of manual skills(1= very bad, 10 = above average)	5.9 (± 1.6)	5.8 (± 1.7)	6.0 (± 1.7)
Self-assessment suture technique (1= very bad, 10 = above average)	3.8 (± 2.3)	3.5 (± 2.2)	4.1 (± 2.4)

Evaluators rated the surgical ability of students. Due to technical difficulties with recording of one of the videos, only 43 students were included. The total score of the ‘See one, do one’ group was statistically significantly better compared to the ‘See one’ + MST-group (14.38 vs. 11.00, *p* < 0.001). The efficiency (1.19 vs. 0.86, *p* = 0.015), handling of instruments (1.31 vs. 0.93, *p* = 0.004), tissue handling (1.60 vs. 1.13, *p* = 0.002), sequence of procedural steps (1.67 vs. 1.34, *p* = 0.010), and the quality of the result (1.33 vs. 1.0, *p* = 0.038) were also statistically significantly better for the conventional group. However, for tissue handling, sewing technique, preparation of the mesh and the skin incision, no statistically significant difference was observed between the two groups (Table 2).

**Table 2:** Surgical skills rated by evaluators on a three-point Likert scale. Mean (± SD) is given and one-way ANOVA with Bonferroni correction was performed

	‘See one, do one’ (mean ± SD) n = 21	‘See one’ + MST(mean ± SD) n = 22	*p*-value
Total score	14.38 (± 2.41)	11.00 (± 3.56)	**<0.001**
Tissue handling	1.29 (± 0.41)	1.25 (± 0.40)	0.773
Efficiency	1.19 (± 0.37)	0.86 (± 0.47)	**0.015**
Speed	1.07 (± 0.40)	0.64 (± 0.52)	**0.004**
Handling of Instruments	1.31 (± 0.33)	0.93 (± 0.52)	**0.007**
Sewing technique	1.24 (± 0.49)	1.05 (± 0.62)	0.264
Preparation of mesh	1.55 (± 0.27)	1.34 (± 0.39)	0.051
Skin incision	1.76 (± 0.46)	1.68 (± 0.42)	0.558
Tissue preparation	1.60 (± 0.41)	1.13 (± 0.49)	**0.002**
Sequence of procedural steps	1.67 (± 0.37)	1.34 (± 0.42)	**0.010**
Quality	1.33 (± 0.50)	1.00 (± 0.51)	**0.038**

The average surgery time for the ‘See one, do one’ group was 43 min (SD = 8 min), while the average time for the ‘See one’ + MST group was 58 min (SD = 14 min). The difference was statistically significant (*p* < 0.001). The conventional group was faster in all sub-steps of the surgery. Both groups’ stitching was consistent with the ‘small stitches, small bites’ rule; no difference in stitching accuracy or consistency was found. More details are listed in Table 3.

**Table 3:** Surgery time and sewing distance. Mean (± SD) is given and one-way ANOVA with Bonferroni correction was performed

	‘See one, do one’ (mean ± SD) *n* = 21	‘See one’ + MST (mean ± SD) (*n* = 22)	*p*-value
Time of simulated surgery (min)	43 (± 8)	58 (± 14)	**< 0.001**
Time for sewing posterior fascia (min)	13 (± 5)	17 (± 7)	**0.023**
Time for sewing anterior fascia (min)	17 (± 5)	24 (± 8)	**0.007**
Average distance between stitches (mm)	5.2 (± 0.93)	5.0 (± 1.03)	0.542
Average distance of the stitches to the fascia margins (mm)	4.7 (± 0.89)	4.5 (± 0.87)	0.393

## Discussion

This study was designed as an exploratory assessment of whether a single-session, low-resource MST protocol might yield measurable benefits for novice learners. Given prior evidence highlighting the need of a structured, multisession mental rehearsal, the limited training effect observed in our study was not unexpected. Nevertheless, the findings offer valuable insight into the feasibility and limitations of minimal MST formats under real-world curricular constraints.

In the light of increasing procedural complexity and evolving demands in surgical training, it is important to evaluated how emerging methods such as MST can be used most effectively [26]. In our study medical students receiving MST alone performed significantly worse than those in the conventional group. This aligns with the findings from Mulla et al., who suggests that MST cannot fully replace conventional training [27]. They tested MST on laparoscopic skills and compared it with a box trainer and a virtual reality simulator. Assessment methods included time, accuracy, precision and performance, in which all the other training methods performed better than MST. In our study, participants in the ‘See one’ + MST group performed significantly worse in most objective measures, such as speed and overall performance. This underlines that, in the current format and for novice learners, MST alone cannot substitute for hands-on experience.

Compared to lecture-based learning or no training at all, MST has been shown to be more effective in several studies [15, 28, 29]. However, in most of these studies, MST was either applied as an adjunct to physical training or involved repeated sessions over time. This might explain why our results differ from those studies with more favourable outcomes. Arora et al. tested the performance of participants on a VR laparoscopic cholecystectomy simulator [29]. They showed that MST performs significantly better than a theoretical lecture for novice surgeons. This latter study used a video-based objective assessment tool, similar to the one used in our study [29]. Studies also suggest that MST mostly plays a role as an additional training tool. It has been proven most effective when coupled with physical training [7, 30].

In this study students only practiced MST for 45 min. Supporting the potential efficacy of short-duration interventions, Lim et al. found that a concise mental imagery training session was sufficient to enhance technical skills in novice anaesthesiology residents [13]. However, many studies suggest that a longer training time of multiple sessions spanning multiple days is more effective [6, 20, 31]. Bathalon et al. have been successful instructing students to practice by themselves at home [30]. After two weeks of individual practice, students were examined. They used an objective structured clinical examination (OSCE) simulation to test performance in emergency situations. In this case, MST coupled with kinesiology training performed significantly better than the standard advanced trauma life support (ATLS) training (20.3 vs. 18.2; *p* < 0.05; max. 25 points) [30]. These findings support the hypothesis that a single MST session, especially without structured feedback or repeated training, is likely insufficient for novice learners to acquire complex procedural skills [18]. In addition to training duration, the structure and content of MST intervention appears to be crucial. The PETTLEP model (Physical, Environment, Task, Timing, Learning, Emotion, and Perspective) has been proposed as a theoretical framework for optimizing mental rehearsal by ensuring that the imagery closely mirrors real-life performance conditions [12]. PETTLEP-based mental practice enhances skill acquisition more effectively than generic visualization techniques [32]. Our MST intervention did not systematically follow the PETTLEP framework, nor did it include feedback, task individualisation, or multisensory stimulation. Thus, it cannot be considered representative of other validated MST protocols.

In our study, the MST protocol was intentionally kept brief and resource efficient. However, this limited scope likely contributed to its reduced impact. Despite differences in time and overall performance, our study showed that both the ‘See one’ + MST and the ‘See one, do one’ group were similarly accurate in their stitching. Considering that most participants gave a low rating to their own suturing skills, it was thought that the ‘See one, do one’ group received a significant advantage by practicing suturing during their physical training. Additionally, MST was not expected to train in suturing, as indicated by Immenroth et al., who showed that MST works well mostly for complex tasks and cognitive aspects [6]. Contrary to our study, Immenroth et al. tested experienced surgeons who had already mastered skills such as suturing [6]. Our findings suggest that for inexperienced students, MST may help with mental preparation for basic skills, but in the format tested here, this effect appears to be minimal. However, as the conventional group performed better in most other aspects, this effect seems to be almost negligible. Germanyuk et al. tested MST against conventional training on medical students practicing wound care [33]. In this case, MST performed similarly well as conventional training, indicating that it might have potential for teaching basic surgical skills if optimally structured.

The MST strategy we used, detailed by Mayer and Hermann, was developed for surgeons trying to optimize their performance [9]. However, its direct application to inexperienced students may have limited effectiveness. MST strategy used for medical students perhaps should have taken a different approach to increase the small training effect we showed [34, 35]. Developing and evaluating an improved MST concept specifically designed for inexperienced students could be the aim of future studies.

Future research should therefore focus on development and validating MST protocols, that are tailored to the needs of medical students, ideally based on PETTLEP principles and embedded in longitudinal training context.

### Surgical training models

Surgical models offer a wide variety of practical training opportunities. They offer a safe and cost effective environment to practice procedures [36]. Different types of models are available. Benchtop models are simple but a less realistic option. The application of this type of model has been validated by previous studies [37, 38]. Commercial benchtop models offer a wide variety of realism and cost [39]. With a combined material price of Less than 5.00 USD the model created for this study is significantly less expensive than most commercially available models [39]. Due to their simplicity, benchtop models are mostly used to train students or residents [40]. Cadavers and animal models are more authentic but are expensive and raise ethical concerns [36, 39]. They are most useful for experienced surgeons to train advanced skills including teamwork and communication [40].

### Limitations

This study is Limited by the relatively small number of participants and the absence of a formal a priori power calculation. Due to the complexity and time-intensive nature of the study, as well as the lack of financial compensation, only a Limited number of students chose to participate. Nevertheless, the final sample size of 44 students is comparable to that of similar studies in the field. Although the small sample size may limit the generalizability of the findings and increases the risk of a type II error, the statistically significant group differences observed suggest that the study was nonetheless sufficiently powered to detect meaningful effects.

Another important Limitation concerns the structure and duration of the MST intervention. While our study employed a single 45-minute session, existing evidence suggests that repeated and spaced MST sessions, ideally grounded in PETTLEP principles, may enhance training effects and long-term retention. Our intervention did not systematically implement the components of the PETTLEP framework, which are considered essential for effective mental rehearsal. Due to curricular constraints and the exploratory nature of this study, a brief, one-time intervention was chosen as a pragmatic and scalable approach within the context of medical education. We acknowledge that this represent a minimalist form of MST, which cannot be considered representative of validated MST protocols. Future studies should consider implementing more comprehensive and longitudinal MST protocols, provided institutional resources and time permit, to further explore the full potential of mental training in surgical education.

A major strength of this study is the simulation and recording of the full procedure, which was a good learning experience for medical students with no previous surgical knowledge. The video examination in our study was conducted by four experienced surgeons, which further increases the validity of the ratings.

## Conclusion

MST, its application for professional surgeons and its effects on surgical performance and stress management, is a well-studied subject. The aim of this study was to explore whether a minimalist MST intervention could support the surgical performance of medical students following observational learning. Our results indicates that the ‘see one’ + MST group performed inferiorly to the ‘see one, do one’ group across several objective performance metrics. This suggest that mental rehearsal alone, at least in the minimal form tested here, may not be sufficient to teach complex surgical skills to medical students. While previous studies indicate that MST could be helpful in combination with ‘See one, do one’ to secure a long-lasting effect, deeper understanding and an effective retention of acquired knowledge and practical skills, our study did not include such a combined intervention. Future research should investigate whether integrating MST, ideally using the PETTLEP Model, into hands-on training protocols can improve learning outcome and resource efficiency in surgical training.

## Supplementary Information

Below is the link to the electronic supplementary material.

Supplementary File 1 (DOCX 15.6 MB)

## Data Availability

The datasets generated and analysed in the current study are not publicly available due to the Ethics Committee restrictions but are available from the corresponding author upon reasonable request.
