# Supplementary material for: Is mental training suitable for teaching a surgical procedure to students? A single-center study using a hernia model
Source: Hernia. 2025 Oct 3;29(1):286. doi: 10.1007/s10029-025-03466-w (PMC12494617; doi:10.1007/s10029-025-03466-w)
Supplement: Supplementary file 1 — (DOCX 15.6 MB) [file 10029_2025_3466_MOESM1_ESM.docx]

Supplementary 1. Two examples of defined key procedural elements from the ‘See One’ and MST Groups. This supplement presents photographs of the identified key procedural elements as documented on the flipchart, accompanied by their English transcription.

| 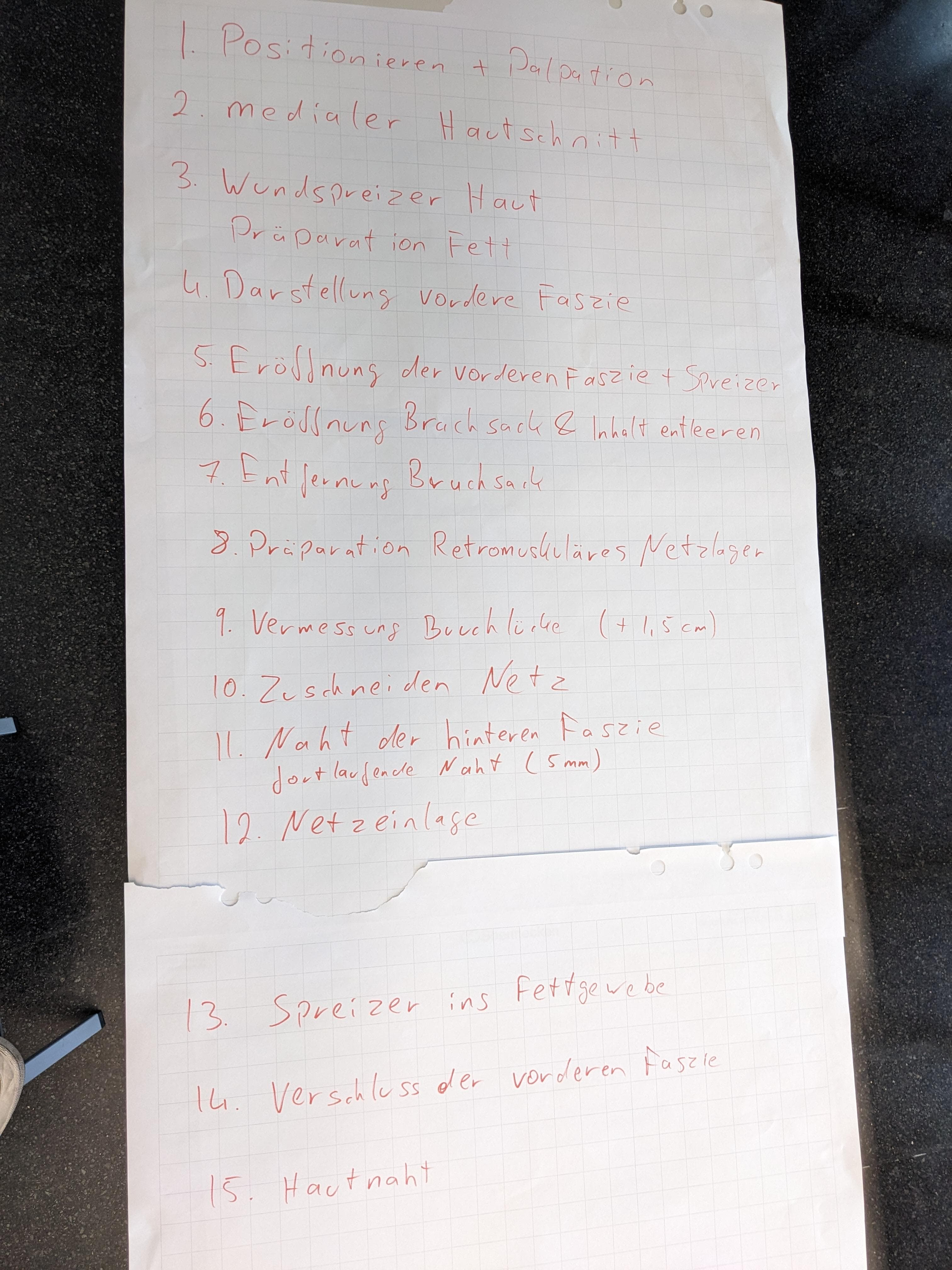 | | 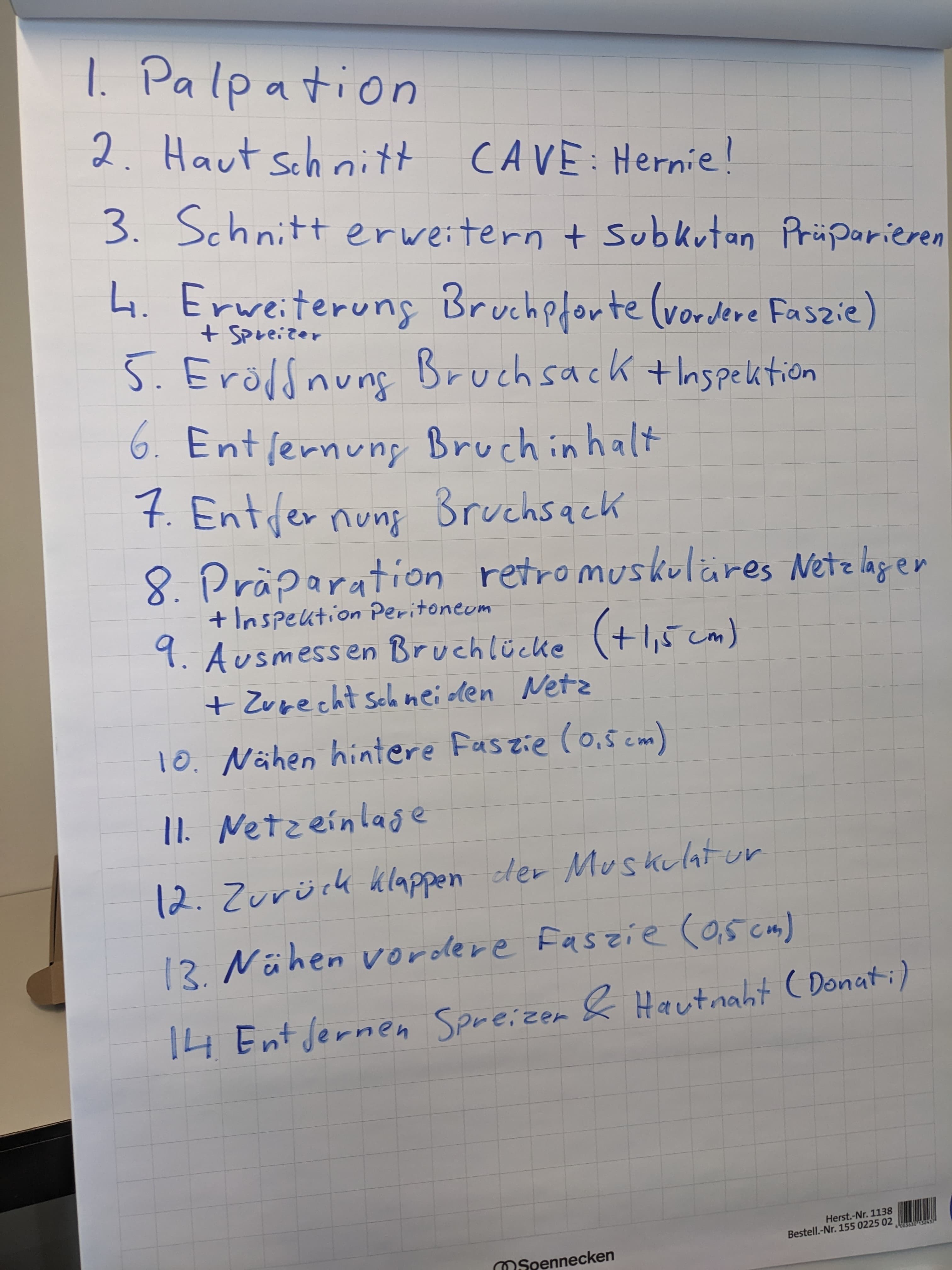 | |
| --- | --- | --- | --- |
| **Transcription of the defined key procedural elementes** | | | |
| **1** | Positioning and palpation | | Palpation |
| **2** | Midline skin incision | | Skin incision – Note: hernia! |
| **3** | Wound retractor in place, dissection of subcutaneous fat | | Extension of incision + subcutaneous dissection |
| **4** | Exposure of the anterior fascia | | Enlargement of the hernia orifice (anterior fascia) + retractor placement |
| **5** | Opening of the anterior fascia + retraction | | Opening of the hernia sac + inspection |
| **6** | Opening of the hernia sac & evacuation of contents | | Removal of hernia contents |
| **7** | Resection of the hernia sac | | Resection of the hernia sac |
| **8** | Preparation of the retromuscular mesh pocket | | Preparation of the retromuscular mesh pocket + inspection of the peritoneum |
| **9** | Measurement of the hernia defect (+1.5 cm) | | Measurement of the hernia defect (+1.5 cm) + tailoring of the mesh |
| **10** | Tailoring the mesh | | Suturing of the posterior fascia (0.5 cm stitch spacing) |
| **11** | Continuous suture of the posterior fascia (5 mm stitch spacing) | | Mesh placement |
| **12** | Mesh placement | | Repositioning of the muscle layer |
| **13** | Retractor placed in subcutaneous tissue | | Suturing of the anterior fascia (0.5 cm stitch spacing) |
| **14** | Closure of the anterior fascia | | Removal of retractors & skin closure (Donati suture) |
| **15** | Skin closure | |  |
